# Telemedicine satisfaction in children with neurological impairment: the role of disease severity, nutritional status, and travel burden

**DOI:** 10.3389/fped.2026.1856840

**Published:** 2026-09-07

**Authors:** Francesca Eletti, Veronica Perico, Eliana Elisabetta Perna, Valeria Calcaterra, Sara Vizzuso, Barbara Borsani, Gianvincenzo Zuccotti

**Affiliations:** 1 Department of Biomedical and Clinical Sciences, University of Milan, Milan, Italy; 2 Department of Pediatrics, Vittore Buzzi Children’s Hospital, Milan, Italy; 3 Pediatric and Adolescent Unit, Department of Internal Medicine, University of Pavia, Pavia, Italy

**Keywords:** caregiver satisfaction, continuity of care, neurological impairment, nutritional status, pediatric neurology, rare neurological diseases, telehealth, telemedicine

## Abstract

**Background:**

Telemedicine is increasingly used in the follow-up of children with neurological impairment to improve access to specialized care and reduce the burden associated with frequent hospital visits. However, data on caregiver satisfaction and the factors influencing perceived quality of telemedicine services in this population remain limited. Therefore, this study aimed to assess caregiver satisfaction with telemedicine consultations delivered as part of a hybrid model combining routine in-person follow-up and video consultations, and to explore its association with nutritional status and other clinical and logistical factors in children with neurological impairment.

**Methods:**

We performed a preliminary analysis of an ongoing observational cross-sectional study including children and adolescents with chronic neurological impairment followed at a tertiary pediatric center through a hybrid model of care combining routine face-to-face follow-up and telemedicine consultations. Caregiver satisfaction with telemedicine was assessed using a structured questionnaire administered after the visit. Clinical, nutritional, and logistical variables were collected, including diagnosis, gastrostomy, BMI *z*-score, quality of life, distance from the hospital, and estimated travel costs. Associations were explored through univariate analyses.

**Results:**

During the study period, 154 patients were evaluated, of whom 65 participated in telemedicine visits and completed the satisfaction questionnaire. Overall, caregiver satisfaction with telemedicine was high, with 93.8% of caregivers reporting high or excellent satisfaction. No significant associations were found between caregiver satisfaction and demographic, clinical, nutritional, or logistical variables, with the exception of a weak inverse association with the PedsQL total score.

**Conclusion:**

Telemedicine represents an effective and well-accepted model of care for children with complex neurological conditions. Caregiver satisfaction was consistently high and did not appear to be influenced by clinical, nutritional, or logistical factors. These findings support the role of telemedicine as a complementary component of a hybrid, family-centered model of care for the long-term management of children with complex neurological conditions, including those requiring multidisciplinary and nutritional follow-up.

## 1 Introduction

Children with chronic and complex neurological conditions require long-term, multidisciplinary follow-up, often delivered in tertiary referral centers. This model of care involves frequent hospital visits and imposes a substantial organizational, economic, and emotional burden on families, particularly for children with motor, cognitive, or sensory impairments who require integrated rehabilitative, nutritional, and psychosocial interventions, often involving multiple healthcare professionals (1). The impact of these conditions extends beyond clinical aspects, significantly affecting family functioning and health-related quality of life (HRQoL), with increased levels of parental stress, reduced social participation, and greater economic burden (1, 2). In this context, HRQoL has emerged as a key outcome in pediatric chronic conditions, reflecting both patient and family well-being (2). Children with neurological disorders, such as cerebral palsy, often experience significantly lower HRQoL compared to healthy peers, particularly in physical and psychosocial domains (3). In recent years, telemedicine has progressively become an integral component of routine clinical practice in neurological care, moving beyond its initial role as an emergency solution. Structured neuro-telehealth models implemented in tertiary neurological centers have demonstrated feasibility and effectiveness in ensuring continuity of care for fragile patients while reducing the need for in-person visits (4). During and after the COVID-19 pandemic, telemedicine use was further consolidated in the management of chronic neurological conditions, as highlighted by multicenter experiences and international recommendations in epilepsy care (5–8). In pediatric populations, telemedicine has been successfully applied across a wide range of clinical disciplines, including epilepsy management, neurological rehabilitation, occupational therapy, speech and language therapy, and the assessment and treatment of sensory and motor disabilities (9–12). In addition, telerehabilitation and mHealth interventions have shown promising results in children with hearing, visual, and motor impairments, improving access to specialized services and supporting continuity of care (13–15). Recent evidence has also highlighted that remote assessment in children with rare and complex neurological disorders is both feasible and clinically valuable, while emphasizing important practical limitations, including the need for caregiver involvement, appropriate technological resources, and careful patient selection. These findings further support the importance of evaluating caregiver perspectives when implementing telemedicine in complex pediatric neurological care (16). The effectiveness of telemedicine has also been demonstrated in geographically disadvantaged and rural settings, where it has improved access to pediatric specialty care and reduced healthcare disparities (17, 18). Alongside clinical and organizational outcomes, caregiver experience represents a key determinant for the sustainable implementation of telemedicine in routine practice. Reduced travel burden, increased flexibility, and improved continuity of care are among the main facilitators reported by caregivers (19, 20). Previous studies conducted in neurological populations have reported high levels of satisfaction with telemedicine among both patients and caregivers, as well as a significant reduction in caregiver burden, highlighting its potential to support more accessible and family-centered models of care (21).

Nevertheless, despite the widespread adoption of telemedicine in pediatric neurology and related fields, data on caregiver-reported satisfaction remain limited, particularly in children with complex neurological impairment and high care needs.

This gap is even more relevant when considering clinical domains that require continuous monitoring and multidisciplinary management. Children with neurological impairment are at high risk of malnutrition and feeding difficulties, which may negatively affect growth, morbidity, and overall quality of life. Feeding and swallowing disorders are highly prevalent in this population and often require long-term nutritional support, including enteral nutrition, as part of comprehensive care (22–24). Nutritional status is closely associated with clinical outcomes and represents a key component of multidisciplinary management in children with neurological disorders (23). However, the integration of nutritional assessment within telemedicine pathways remains poorly explored. In particular, it is still unclear whether telemedicine can adequately support the complex nutritional needs of children with neurological impairment and how caregiver perception of telemedicine may be influenced by nutritional and clinical burden. In this context, telemedicine may represent a valuable opportunity to deliver integrated, multidisciplinary, and family-centered care while reducing the burden associated with frequent hospital visits. Therefore, understanding caregiver satisfaction is essential to determine whether telemedicine can be sustainably integrated into multidisciplinary nutritional care pathways for children with neurological impairment while reducing the burden associated with frequent hospital visits.

The aim of the present study was to evaluate caregiver satisfaction with telemedicine consultations delivered as part of a hybrid follow-up model combining routine face-to-face care and video consultations in children with neurological impairment, and to explore factors associated with perceived satisfaction, with particular attention to travel-related burden, clinical characteristics and nutritional status, in order to inform the future development and optimization of patient-centered telemedicine care pathways.

## 2 Material and methods

### 2.1 Study design and participants

This observational, cross-sectional study was conducted at a tertiary pediatric referral center, *“Vittore Buzzi” Children's Hospital*, Milan, Italy, to evaluate caregiver satisfaction with telemedicine and its association with clinical, nutritional, and logistical factors in children with neurological impairment. This study reports a preliminary analysis of an ongoing observational study. Data were collected between January 2024 and December 2025 as part of routine clinical care. All caregivers of children and adolescents followed at the center for chronic neurological impairment were invited to participate. Eligible patients were males and females aged 9 months to 18 years with a diagnosis of neurological impairment, including neuromuscular diseases, neurodegenerative diseases, genetic syndromes, encephalopathies, and cerebral palsy. Both newly diagnosed patients and those already receiving follow-up at the center were eligible for inclusion. To assess the potential for selection bias, baseline demographic and clinical characteristics of patients included in the telemedicine satisfaction analysis were compared with those of patients who received standard in-person follow-up only.

Clinical and demographic data were collected from medical records at the baseline in-person visit. Diagnoses were grouped into five main categories: encephalopathies (including cerebral palsy), neuromuscular diseases, neurodegenerative diseases, genetic syndromes, and neurometabolic diseases. Nutritional status was assessed using BMI-for-age *z* scores according to World Health Organization (WHO) growth standards (25) for children under 2 years of age and Centers for Disease Control and Prevention (CDC) growth charts for older children (26).

For the present analysis, only patients who underwent at least one telemedicine consultation were eligible for the caregiver satisfaction analysis, as the satisfaction questionnaire was administered exclusively following telemedicine visits. All eligible caregivers completed the questionnaire; therefore, no participants were excluded because of incomplete questionnaire data.

### 2.2 Telemedicine visits

All patients underwent an initial in-person clinical and nutritional assessment, followed by routine follow-up based on a hybrid care model combining in-person visits and video consultations. Telemedicine consultations were offered in addition to standard face-to-face care and were intended to complement, rather than replace, routine clinical follow-up. The decision to schedule a telemedicine consultation was based on clinical judgment and organizational considerations, including the patient's clinical condition and family's needs, without predefined standardized criteria. When considered appropriate, telemedicine visits were generally scheduled every 2–3 months, while regular in-person assessments continued according to routine clinical practice.

Telemedicine visits were integrated into standard clinical care and conducted by the multidisciplinary team. Consultations were performed using secure, institution-approved video conferencing platforms (COD20) (27), and involved the healthcare professionals responsible for the patient's care. Telemedicine appointments focused on clinical follow-up, caregiver counseling, and monitoring of disease-related aspects, including functional status and nutritional management when indicated. Caregiver satisfaction was assessed only among caregivers who had participated in at least one telemedicine consultation and completed the satisfaction questionnaire.

### 2.3 Assessment of caregiver satisfaction with telemedicine

Caregiver-reported satisfaction with telemedicine was assessed using a structured questionnaire specifically developed for this study by adapting domains from previously published telemedicine satisfaction instruments, including the Telemedicine Satisfaction Questionnaire (TSQ) (28) the telehealth Usability Questionnaire (TUQ) (29), and the Digital Health Acceptability Questionnaire (DHAQ) (30). Rather than reproducing any of these instruments in their original form, selected domains were adapted to the objectives of the present study and to the pediatric neurological setting. Items were selected and reworded to reflect the specific characteristics of pediatric neurological follow-up, while preserving the original domains related to technical quality, usability, communication, and overall satisfaction. The questionnaire was administered within 3 days after the telemedicine visit in order to minimize recall bias and ensure consistency in data collection. The questionnaire included 10 items evaluating different aspects of the telemedicine experience, including technical quality (audio quality, visual quality, ease of connection), consultation experience (personal comfort, privacy during the consultation, duration of the consultation), communication with the healthcare professional (clarity of treatment explanation, perceived accuracy of the specialist, satisfaction with the specialist), and overall telemedicine experience. The complete questionnaire is provided as Supplementary Material 1. Each item was rated on a 4-point Likert scale (1 = poor, 2 = moderate, 3 = good, 4 = excellent). These domains were selected because they represent the principal dimensions commonly assessed in telemedicine satisfaction instruments including technical quality, usability, communication with the healthcare professional, and overall consultation experience. Caregivers were also asked to report their place of residence, the one-way distance (km) between their residence and the hospital, and the estimated travel cost usually incurred to attend an in-person hospital visit. Travel costs were self-reported by caregivers as an overall estimate and were not further categorized into specific expense components. A total satisfaction score was calculated by summing the scores of the 10 questionnaire items, resulting in a possible score ranging from 10 to 40, with higher scores indicating greater caregiver satisfaction. For descriptive purposes, total scores were grouped into four predefined categories: low satisfaction (10–19), moderate satisfaction (20–29), good satisfaction (30–35), and excellent satisfaction (36–40). Additional questions explored willingness to use telemedicine again and to recommend telemedicine to others. Although the questionnaire was informed by previously published telemedicine satisfaction instruments, it was not formally validated in this specific pediatric neurological population. Nevertheless, the adapted questionnaire demonstrated good internal consistency in the present cohort (Cronbach's *α* = 0.859). Therefore, the questionnaire should be considered an exploratory instrument developed for this preliminary study. The total satisfaction score was analyzed descriptively together with the individual questionnaire items. Individual questionnaire items were analyzed descriptively and expressed as mean and standard deviation. Correlations between individual questionnaire items and overall satisfaction were assessed using Spearman correlation coefficients.

### 2.4 Data collection

Clinical and demographic data were collected from medical records, including patient age, sex, diagnosis, presence of gastrostomy (percutaneous endoscopic gastrostomy, PEG), and nutritional status. Information on travel-related burden, including distance from the hospital and estimated travel costs for in-person visits was self-reported by caregivers through the telemedicine questionnaire. The caregiver satisfaction questionnaire was administered within 3 days after the telemedicine consultation, as described above, and responses were recorded using the study identification code. Questionnaire data were collected using study identification codes and stored in a secure REDCap database accessible only to authorized study personnel. Personal identifiers were not included in the analytical dataset, and all analyses were performed on pseudonymized data. Quality of life was assessed using PedsQL 3.0 Cerebral Palsy Module parent-report (3). Although originally developed for children with cerebral palsy, this instrument was used in the present study as a proxy measure of functional impairment and caregiver-perceived quality of life because many neurological conditions included in the cohort share similar motor and functional limitations. In this study, higher scores indicate greater impairment and lower quality of life.

### 2.5 Nutritional status assessment

Nutritional status was assessed using anthropometric data collected during routine clinical care. Anthropometric measurements were obtained using standardized procedures during routine in-person clinical assessments. Body weight was measured using a calibrated scale. In patients with severe motor impairment who were unable to stand, weight was obtained using a wheelchair scale when available, or by subtracting caregiver weight when measured together. Height was measured using a stadiometer in patients able to stand. In patient unable to stand, height was estimated using segmental measurements (knee-heel length) according to Stevenson's method, using standard predictive equations (31). For telemedicine consultations, when updated anthropometric data were required, caregivers were asked to report the child's body weight measured on the day of the consultation and to perform a knee-heel length measurement at home following instructions provided by the clinical team. Height was estimated from the knee-heel length using Stevenson's method (31). BMI was calculated as body weight (kg) divided by estimated height squared (m^2^), and BMI-for-age *z*-scores were subsequently calculated according to the applicable growth references. Body mass index (BMI)-for-age *z*-scores were calculated according to WHO growth standards for children under 2 years of age (32) and CDC growth charts for older children (26). BMI *z*-scores were calculated for all patients to ensure consistency across the study population. In children under 2 years of age, BMI *z*-scores were interpreted with caution, as weight-for-length is generally preferred in this age group. Nutritional status was classified according to BMI *z*-score as follows: severe malnutrition (*z*-score < −3), moderate malnutrition (*z*-score between −3 and −2), risk of malnutrition (*z*-score between −2 and −1), normal weight (*z*-score between −1 and +1), and overweight (*z*-score >+1).

### 2.6 Statistical analysis

Continuous variables were expressed as mean ± standard deviation, and categorical variables as number and percentage. Normality was assessed using the Shapiro–Wilk test. Parametric or non-parametric statistical tests were selected as appropriate according to data distribution.

The primary outcome of the study was caregiver satisfaction with telemedicine, expressed as the total satisfaction score. Clinical, nutritional, demographic, and logistical variables (age, diagnosis, BMI *z*-score, PedsQL score, PEG, distance from the hospital, and travel costs) were evaluated as potential factors associated with caregiver satisfaction using univariate analyses only. Correlations between individual telemedicine questionnaire items and the overall satisfaction score were assessed using Spearman correlation coefficients. A *p*-value <0.05 was considered statistically significant. Statistical analyses were performed using JMP software and R (version 4.6.0).

### 2.7 Ethics statement

This study was approved by the local Ethics Committee (Lombardia 1, protocol number 81-2024) and conducted in accordance with the principles of the Declaration of Helsinki. Written informed consent was obtained from the parents or legal guardians of all participating children after full information on the nature of the study had been provided. All collected data were pseudonymized and collected in a database established on the REDCap platform (https://www.project-redcap.org), hosted by Harvard Catalyst, Boston, USA, in strict compliance with applicable GDPR regulations.

## 3 Results

### 3.1 Study population

**Figure 1:**
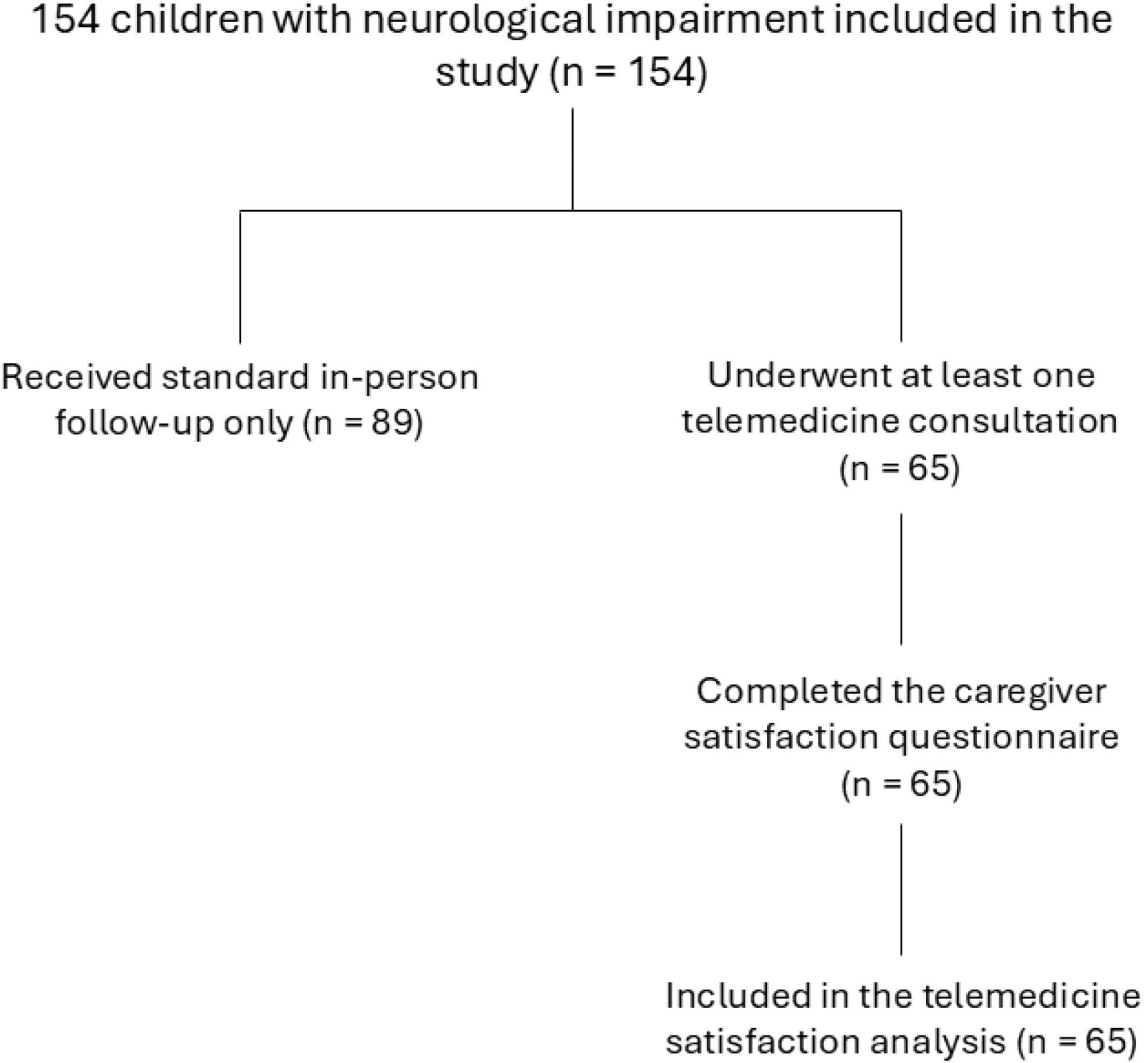
Flowchart illustrating the selection of participants included in the caregiver satisfaction analysis. Among the 154 children with neurological impairment included in the study, 65 underwent at least one telemedicine consultation and completed the caregiver satisfaction questionnaire, whereas 89 received standard in-person follow-up only.

During the study period, a total of 154 children with neurological impairment were evaluated at the center. The mean age was 13.2 ± 3.9 years, and 56.9% of patients were fed via gastrostomy. The mean BMI *z*-score was −2.29 ± 2.86, with 51.8% of the cohort classified as moderately or severely malnourished. The most represented diagnostic groups were encephalopathies (including cerebral palsy), neuromuscular diseases, and genetic syndromes. The mean PedsQL total score was 76.54 ± 21.45. Detailed characteristics of the overall study cohort (*n* = 154) are reported in Supplementary Table 1. Among the overall cohort, 65 patients underwent at least one telemedicine consultation and were therefore eligible for the caregiver satisfaction analysis. The participant flow and selection for the telemedicine satisfaction analysis are shown in Figure 1.

Baseline characteristics of patients included in the telemedicine satisfaction analysis and those who received standard in-person follow-up only are summarized in Supplementary Table 2. Patients in the telemedicine group tended to be younger (7.6 ± 4.6 vs. 9.1 ± 4.8 years, *p* = 0.059). They also had a higher proportion of female (45% vs. 28%, *p* = 0.034), and a higher prevalence of PEG (58% vs. 38%, *p* = 0.020), whereas no significant differences were observed in diagnosis group (*p* = 0.070) or baseline BMI *z*-score (*p* = 0.269). The demographic, clinical, nutritional, and logistical characteristics of this telemedicine subgroup are summarized in Table 1. The remaining 89 patients received routine in-person follow-up only during the study period and were therefore not eligible to complete the telemedicine satisfaction questionnaire.

**Table 1:** Characteristics of patients who underwent at least one telemedicine consultation and were included in the caregiver satisfaction analysis (*n* = 65). Continuous variables are presented as mean ± SD, while categorical variables are presented as number and percentage.

Demographic and clinical variables	TLM subgroup
Age (years)	7.6 ± 4.6
Sex (Male/Female)	35 (53.8%)/30 (46.2%)
PEG (Yes/No)^a^	37 (57.8%)/27 (42.2%)
BMI *z*-score	−2.69 ± 3.03
PedsQL total score	78.70 ± 19.13
Nutritional status^b^	
Normal	15 (23.8%)
Moderate malnutrition	13 (20.6%)
Severe malnutrition	22 (34.9%)
At risk of malnutrition	12 (19%)
Overweight	1 (1.6%)
Diagnosis group	
Encephalopathy (including PCI)	21 (32.3%)
Genetic syndromes	19 (29.2%)
Neuromuscular diseases	17 (26.1%)
Neurodegenerative diseases	8 (12.3%)
Logistical factors	
Distance from hospital (km)	61.07 ± 116.9
Travel costs (€)	33.38 ± 40.76

a PEG data were available for 64 of the 65 patients.

b Nutritional status was available for 63 patients.

### 3.2 Telemedicine subgroup

The demographic, clinical, nutritional, and logistical characteristics of the telemedicine subgroup are summarized in Table 1.

The mean age was 7.6 ± 4.6 years, and 53.8% were male. Overall, 57.8% of patients had gastrostomy (PEG). The mean BMI *z*-score was −2.69 ± 3.03. Among the 63 patients with available nutritional data, 22 (34.9%) were severely malnourished, 13 (20.6%) were moderately malnourished, 12 (19.0%) were at risk of malnutrition, 15 (23.8%) had normal nutritional status, and 1 (1.6%) was overweight. The most represented diagnostic groups were encephalopathies including cerebral palsy (32.3%) and genetic syndromes (29.2%). The mean distance from the hospital was 61.07 ± 116.9 km, and the mean travel cost was 33.38 ± 40.76 euros. The mean PedsQL total score was 78.70 ± 19.13.

### 3.3 Telemedicine satisfaction

**Figure 2:**
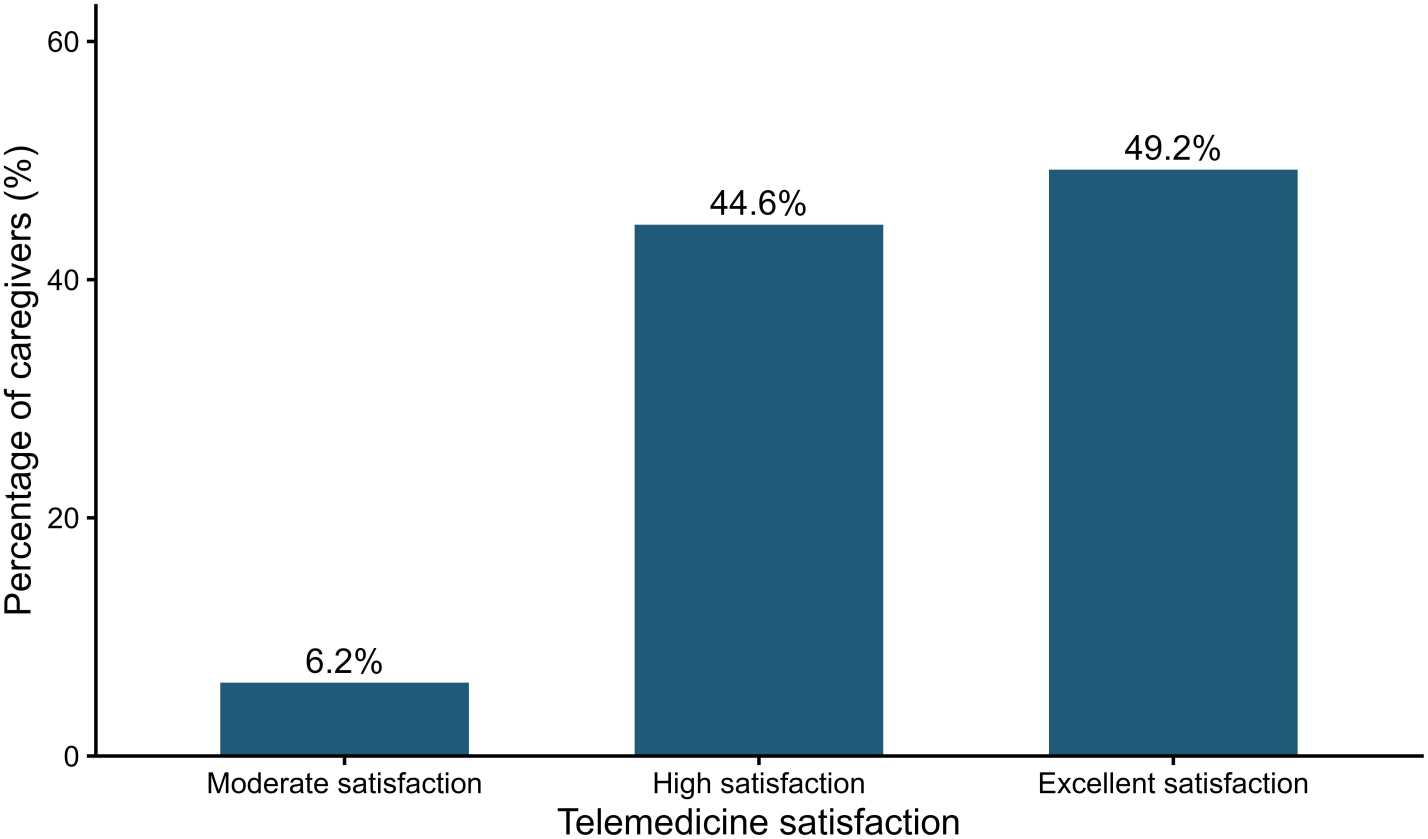
Distribution of caregiver satisfaction with telemedicine. Bars represent the percentage of caregivers reporting moderate, high, and excellent satisfaction (*n* = 65).

Caregiver satisfaction was assessed using the structured questionnaire described in the Methods section, with total scores categorized into four predefined satisfaction levels. Telemedicine satisfaction was generally high. No caregivers reported low satisfaction, while 4 (6.2%) reported moderate satisfaction, 29 (44.6%) high satisfaction, and 32 (49.2%) excellent satisfaction. Overall, 93.8% of caregivers reported high or excellent satisfaction (Figure 2).

### 3.4 Factors associated with telemedicine satisfaction

**Figure 3:**
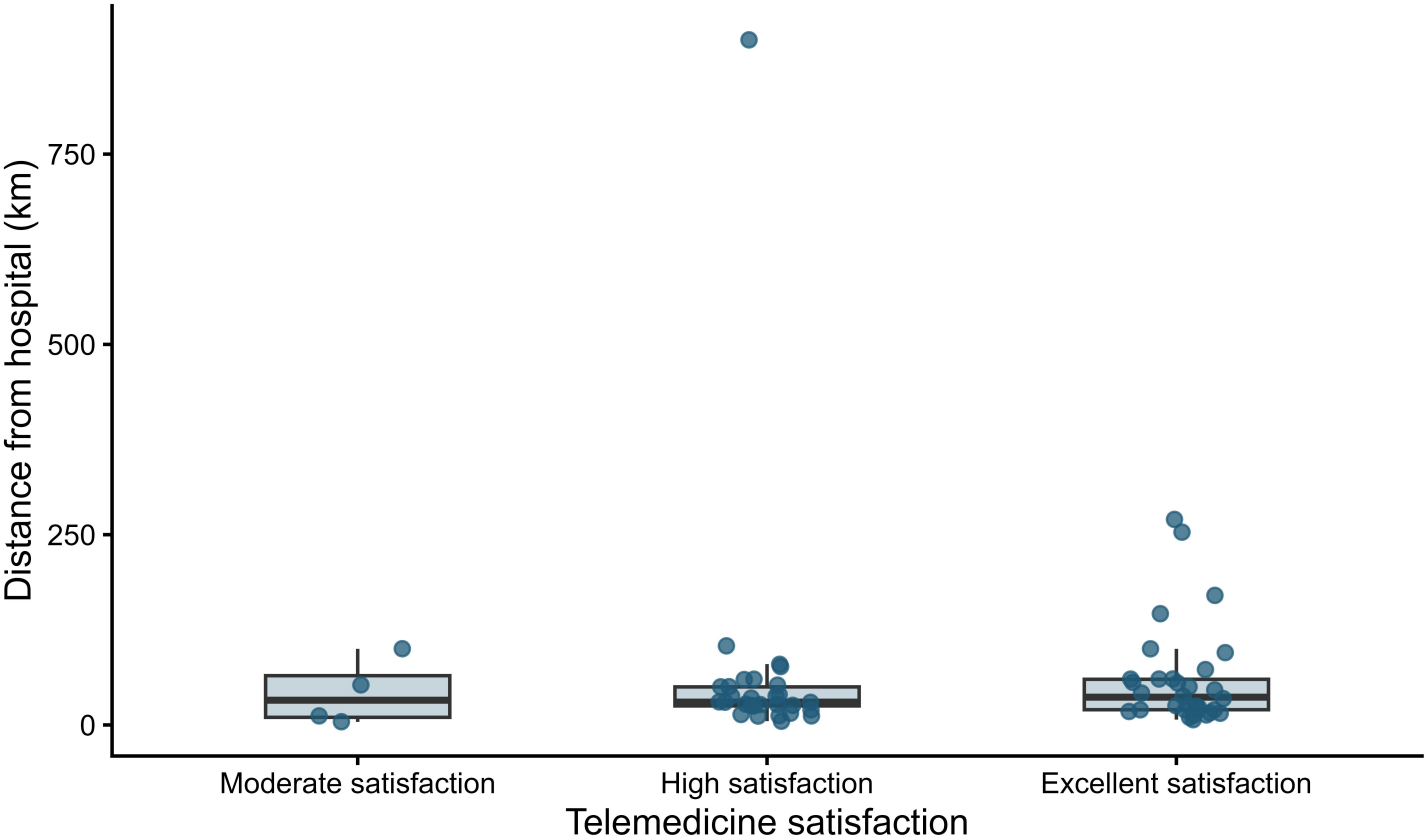
Distribution of travel distance according to caregiver satisfaction. Points represent individual participants; boxes indicate the median and interquartile range (IQR).

**Figure 4:**
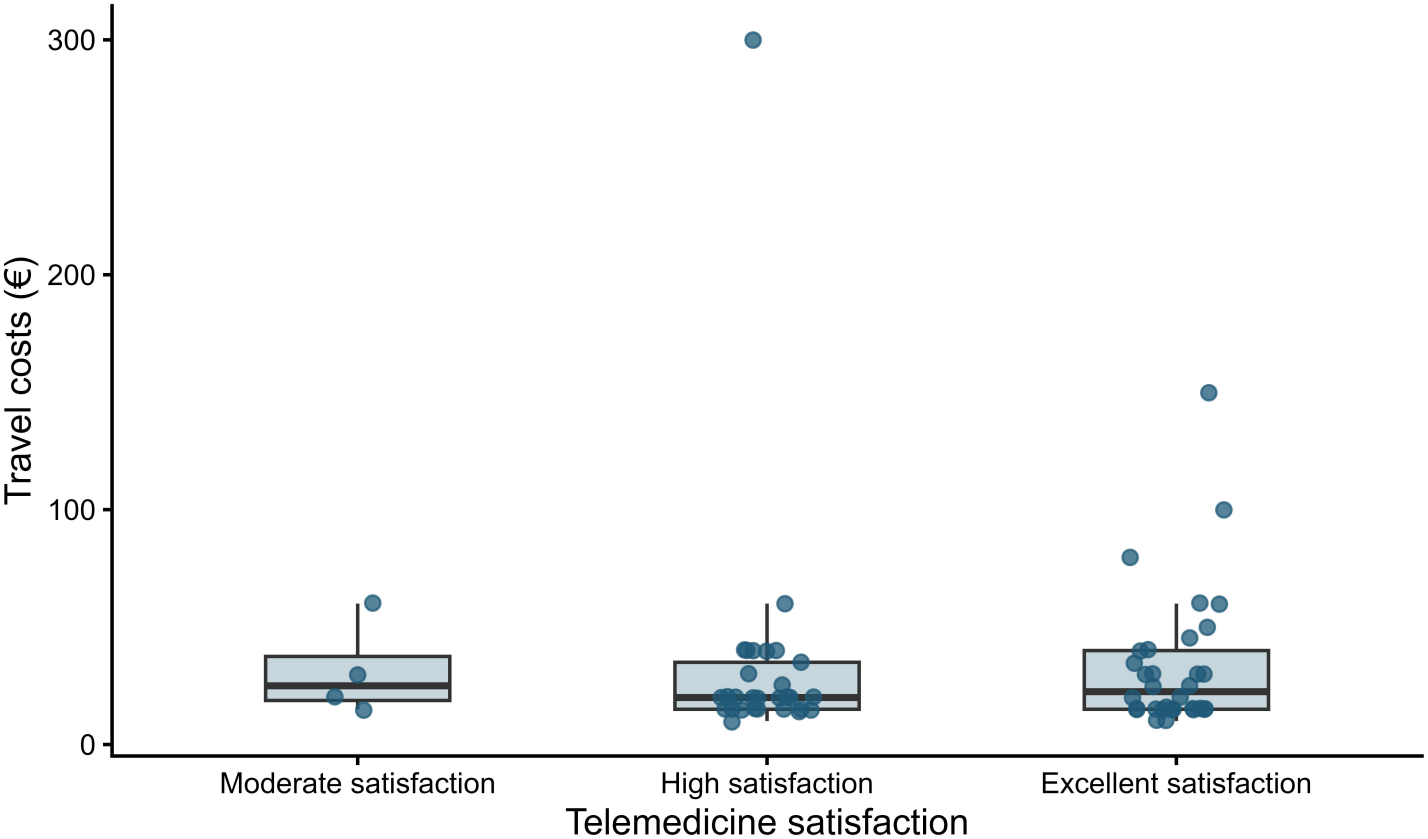
Travel costs according to caregiver satisfaction. Points represent individual participants; boxes indicate the median and interquartile range (IQR).

In the univariate analysis using the continuous total satisfaction score, caregiver satisfaction was not significantly associated with age (Spearman's rho = −0.176, *p* = 0.161), BMI *z*-score (rho = −0.130, *p* = 0.312), travel distance (rho = 0.035, *p* = 0.784), travel cost (rho = −0.015, *p* = 0.905), PEG status (*p* = 0.791), GMFCS severity (*p* = 0.252), or diagnosis group (Kruskal–Wallis, *p* = 0.79). A weak negative correlation was observed between caregiver satisfaction and PedsQL total score (Spearman's rho = −0.246, *p* = 0.049). As shown in Figure 3, the distribution of distance from the hospital was broadly similar across the different satisfaction categories, with substantial overlap between groups despite a few outlying values. Likewise, Figure 4 shows a comparable distribution of travel costs across satisfaction levels, without any clear trend indicating higher or lower satisfaction according to travel expenditure.

### 3.5 Telemedicine satisfaction questionnaire items

Caregiver responses to individual telemedicine questionnaire items showed high level of satisfaction across all domains, with mean scores ranging from 3.13 to 3.75 (Supplementary Table 3). The highest scores were reported for communication with the specialist (3.75 ± 0.43), ease of connection (3.36 ± 0.71), and audio-visual quality of the consultation (3.13 ± 0.68). Correlation analysis showed that overall telemedicine satisfaction was most strongly associated with visual quality of the consultation (*r* = 0.60), satisfaction with the specialist (*r* = 0.56), ease of connection (*r* = 0.53), and perceived competence of the specialist (*r* = 0.47). Detailed results are reported in Supplementary Table 4.

## 4 Discussion

The main finding of this study is that caregiver satisfaction with telemedicine was very high among families of children with neurological impairment. Most caregivers reported high or excellent satisfaction, confirming that telemedicine is a well-accepted model of care in this population. These findings are consistent with previous studies reporting high levels of caregiver satisfaction with telemedicine in pediatric neurological populations, with satisfaction rates ranging from 80% to over 90% (7, 8, 33).

Interestingly, telemedicine satisfaction was not associated with distance from the hospital or travel costs. This suggests that telemedicine may be beneficial not only for families living far from healthcare centers, but also more broadly across different patient groups. In this context, telemedicine may contribute to reducing the daily burden associated with repeated hospital visits and complex care management.

Previous studies have shown that telemedicine improves access to specialized neurological care and reduces the need for in-person visits, particularly in patients with complex chronic conditions (17, 34–36). Another important finding of this study is that technical quality and communication with the specialist were aspects most strongly associated with caregiver satisfaction. With the exception of a weak inverse correlation between caregiver satisfaction and PedsQL total score, no statistically significant associations were observed between caregiver satisfaction and the evaluated demographic, clinical, nutritional, or logistical variables in the univariate analyses. Although caregiver satisfaction was weakly associated with PedsQL total score, no multivariable regression analysis was performed because the highly skewed distribution of caregiver satisfaction (61 caregivers with high/excellent satisfaction vs. 4 with moderate satisfaction) would have resulted in unstable and non-informative model estimates. Since higher PedsQL scores in the present study indicate greater functional impairment and poorer quality of life, this finding suggests that caregivers of children with greater disease burden reported slightly higher satisfaction with telemedicine, although the strength of this association was weak. This suggests that perceived quality of care in telemedicine may be influenced by interactional and technological factors, rather than by organizational aspects such as visit duration. Ensuring good audio-visual quality and effective communication is therefore essential to optimize telemedicine services. These aspects may play a crucial role in shaping caregiver trust and engagement in remote care pathways. Quality of life represents a central outcome in children with neurological impairment and provides information on perceived health status and functional well-being (2, 3). The caregiver satisfaction questionnaire used in this study was specifically developed by adapting domains from previously published telemedicine satisfaction instruments to better reflect the characteristics of pediatric neurological care. Although it was not formally validated in this specific population, it allowed a structured assessment of the main dimensions of the telemedicine experience and should therefore be interpreted as an exploratory measure of caregiver satisfaction.

In our study, no significant associations were observed between telemedicine satisfaction and clinical or nutritional variables. This suggests that telemedicine is well accepted across patients with different levels of clinical complexity and nutritional status. Children with neurological impairment are at high risk of malnutrition and frequently require regular nutritional monitoring and multidisciplinary follow-up (23, 37, 38). Feeding and swallowing difficulties are very common in children with neurological impairment and represent one of the main causes of malnutrition in this population (39–41). Several studies have shown that the risk of malnutrition increases with the severity of neurological impairment and functional limitations (42, 43). Nutritional management, including enteral nutrition and gastrostomy feeding, plays a key role in improving clinical outcomes in children with severe neurological impairment (44, 45). In this context, telemedicine may facilitate regular monitoring and timely nutritional interventions, although these potential benefits were not directly assessed in the present study. The absence of significant associations in the univariate analyses may be related to the relatively small sample size and the overall high level of caregiver satisfaction observed across the cohort. The limited variability and ceiling effect observed in caregiver satisfaction scores may have reduced the ability to detect differences between subgroups.

This study has several limitations. First, this was a single center study with a relatively small sample size, which may limit the generalizability of the findings. Second, satisfaction was assessed using a caregiver-reported questionnaire and may therefore be influenced by subjective perception and response bias. Third, the cross-sectional design does not allow causal inferences. In addition, the study represents a preliminary analysis of an ongoing project, and a larger sample size may allow more robust analyses in the future. Another important limitation is the potential for selection bias, as the caregiver satisfaction analysis was restricted to patients who underwent at least one telemedicine consultation. Since telemedicine consultations were offered according to clinical judgment and organizational considerations rather than predefined allocation criteria, the telemedicine subgroup may not be fully representative of the overall cohort. An additional limitation is the limited variability of caregiver satisfaction scores, with almost all caregivers reporting high or excellent satisfaction. This reduced the ability to detect statistically significant associations between caregiver satisfaction and the evaluated demographic, clinical, nutritional, and logistical variables. Caregiver satisfaction was assessed within the clinical setting by a physician, which may have introduced social desirability bias despite the confidential handling and pseudonymization of questionnaire responses. Consequently, caregivers may have been more inclined to provide favorable evaluations of the telemedicine service. Furthermore, travel costs were self-reported by caregivers as an overall estimate and were not based on objective cost assessments or detailed itemization of individual expenses. Despite these limitations, the study reflects real-life clinical practice in a multidisciplinary pediatric neurological population and provides meaningful insights into caregiver experience with telemedicine in a complex care setting. Future longitudinal studies are needed to determine whether telemedicine follow-up may also contribute to improving clinical outcomes, including nutritional status and quality of life, over time in this population.

## 5 Conclusion

Telemedicine represents a well-accepted and clinically valuable model of care for children with neurological impairment. Caregiver satisfaction was consistently high across the study population, and was not significantly associated with evaluated demographic, clinical, nutritional, or logistical variables with the exception of a weak inverse association with PedsQL total score. However these findings should be interpreted with caution because of the limited variability of satisfaction scores, which reduced the ability to detect potential associations. Nevertheless, the overall high level of caregiver satisfaction supports the feasibility and acceptability of telemedicine as part of multidisciplinary follow-up, including nutritional monitoring. Telemedicine should therefore be considered not only as a tool to improve access to care, but also as a key component of patient-centered care models aimed at supporting continuity of care and reducing family burden. Further longitudinal studies involving larger and more heterogeneous populations are needed to better define the determinants of caregiver satisfaction and the role of telemedicine in long-term care pathways for children with neurological impairment.

## Data Availability

The raw data supporting the conclusions of this article will be made available by the authors, without undue reservation.
