# Supplementary material for: Telemedicine satisfaction in children with neurological impairment: the role of disease severity, nutritional status, and travel burden
Source: Front Pediatr. 2026 Sep 7;14:1856840. doi: 10.3389/fped.2026.1856840 (PMC13590733; doi:10.3389/fped.2026.1856840)
Supplement: Supplementary file 1 [file Table1.docx]

| **Supplementary table 1 - Characteristics of the overall study cohort (n = 154)** |  |
| --- | --- |
| **Variable** | **Total sample** |
| Age (years) | 13.2 ± 3.9 |
| Sex (M/F) | 97 (62.9%) / 57 (37.0%) |
| PEG* (Y/N) | 71 (46.4%) / 82 (53.5%) |
| BMI z-score | −2.29 ± 2.86 |
| **Nutritional status** |  |
| Normal | 41 (26.9%) |
| Moderate malnutrition | 29 (19.0%) |
| Severe malnutrition | 50 (32.8%) |
| At risk of malnutrition | 26 (17.0%) |
| Overweight | 4 (2.6%) |
| Obesity | 2 (1.3%) |
| **Diagnosis group** |  |
| - Encephalopathy (including PCI) | 54 (35.3%) |
| - Genetic syndromes | 30 (19.6%) |
| - Neuromuscular diseases | 38 (24.8%) |
| - Neurodegenerative diseases | 23 (15.0%) |
| - Neurodevelopmental disorders | 1 (0.7%) |
| - Neurometabolic diseases | 6 (3.9%) |
| PedsQL total score | 76.54 ± 21.45 |

Table 1: Data are presented as mean ± standard deviation for continuous variables and number (percentage) for categorical variables. BMI: body mass index; PEG: percutaneous endoscopic gastrostomy; PedsQL: Pediatric Quality of Life Inventory

*PEG status was available for 153 patients
