# Supplementary material for: Telemedicine satisfaction in children with neurological impairment: the role of disease severity, nutritional status, and travel burden
Source: Front Pediatr. 2026 Sep 7;14:1856840. doi: 10.3389/fped.2026.1856840 (PMC13590733; doi:10.3389/fped.2026.1856840)
Supplement: Supplementary file 2 [file Table2.docx]

**Supplementary table 2. Baseline characteristics of patients included and not included in the telemedicine satisfaction analysis**

| Characteristic | No telemedicine  N = 89^1^ | Telemedicine  N =65^1^ | p-value^2^ |
| --- | --- | --- | --- |
| **Age (y)** | 9.1 ± 4.8 | 7.6 ± 4.6 | 0.059 |
| **Sex** |  |  | 0.034 |
| F | 25 (28%) | 29 (45%) |  |
| M | 64 (72%) | 36 (55%) |  |
| **Diagnosis** |  |  | 0.070 |
| Cerebral palsy | 9 (10%) | 8 (12%) |  |
| Encephalopathies | 24 (27%) | 13 (20%) |  |
| Genetic Syndrome | 12 (13%) | 18 (28%) |  |
| Neurodegenerative diseases | 16 (18%) | 8 (12%) |  |
| Neurometabolic diseases | 6 (6.7%) | 0 (0%) |  |
| Neuromuscular diseases | 21 (24%) | 17 (26%) |  |
| Other | 1 (1.1%) | 1 (1.5%) |  |
| **PEG (yes/no)** | 34 (38%) | 38 (58%) | 0.020 |
| **BMI z-score** | -2.02 ± 2.72 | -2.69 ± 3.03 | 0.3 |
|  |  |  |  |

^1^Mean ± SD; n (%)

^2^Wilcoxon rank sum test; Pearson’s Chi-squared test; Fisher’s exact test
