# Supplementary material for: Telemedicine satisfaction in children with neurological impairment: the role of disease severity, nutritional status, and travel burden
Source: Front Pediatr. 2026 Sep 7;14:1856840. doi: 10.3389/fped.2026.1856840 (PMC13590733; doi:10.3389/fped.2026.1856840)
Supplement: Supplementary file 3 [file Table3.docx]

**Supplementary Table 3 – Descriptive statistics of the individual items included in the telemedicine satisfaction questionnaire**

| **Item** | **Mean** | **SD** |
| --- | --- | --- |
| Call quality | 3.16 | 0.65 |
| Video quality | 3.13 | 0.68 |
| Ease of connection | 3.36 | 0.71 |
| Personal comfort | 3.46 | 0.58 |
| Privacy | 3.55 | 0.53 |
| Duration of the appointment | 3.32 | 0.53 |
| Explanation of the treatment | 3.69 | 0.46 |
| Specialist’s expertise | 3.66 | 0.47 |
| Satisfaction with the specialist | 3.75 | 0.43 |
| Overall experience | 3.53 | 0.61 |
|  |  |  |
