# Supplementary material for: Telemedicine satisfaction in children with neurological impairment: the role of disease severity, nutritional status, and travel burden
Source: Front Pediatr. 2026 Sep 7;14:1856840. doi: 10.3389/fped.2026.1856840 (PMC13590733; doi:10.3389/fped.2026.1856840)
Supplement: Supplementary file 4 [file Table4.docx]

**Supplementary Table 4 – Correlation between individual telemedicine questionnaire items and overall telemedicine satisfaction**

| **Item** | **Spearman’s correlation (r)** |
| --- | --- |
| Video quality | 0.60 |
| Ease of connection | 0.53 |
| Personal Comfort | 0.43 |
| Privacy | 0.41 |
| Consultation duration | 0.22 |
| Explanation of the treatment | 0.31 |
| Specialist accuracy | 0.47 |
| Satisfaction with specialist | 0.56 |
| Audio quality | 0.43 |

Supplementary Table 4: Spearman's rank correlation coefficients (r) between each questionnaire item and the total telemedicine satisfaction score.
