# Supplementary material for: Telemedicine satisfaction in children with neurological impairment: the role of disease severity, nutritional status, and travel burden
Source: Front Pediatr. 2026 Sep 7;14:1856840. doi: 10.3389/fped.2026.1856840 (PMC13590733; doi:10.3389/fped.2026.1856840)
Supplement: Supplementary file 5 [file Table5.docx]

# **Telemedicine Satisfaction Questionnaire**

**Initials:** ____________________________  **Sex:** ____________________________

**Age:** ________________________________  **Marital Status:** __________________

**Education Level:** ____________________  **Language Spoken at Home:** ___________

**Place of Residence:** ________________  **Distance from the Hospital:** __________

**Travel Cost:** ________________________  **Diagnosis:** _________________________

**Date of First Visit:** __________________

**Date of Most Recent Visit:** ____________

### Satisfaction Rating

| Item | Poor (1) | Fair (2) | Good (3) | Excellent (4) |
| --- | --- | --- | --- | --- |
| Voice quality | ☐ | ☐ | ☐ | ☐ |
| Video quality | ☐ | ☐ | ☐ | ☐ |
| Ease of connection | ☐ | ☐ | ☐ | ☐ |
| Personal comfort | ☐ | ☐ | ☐ | ☐ |
| Privacy during the consultation | ☐ | ☐ | ☐ | ☐ |
| Duration of the consultation | ☐ | ☐ | ☐ | ☐ |
| Explanation of the treatment | ☐ | ☐ | ☐ | ☐ |
| Specialist’s competence | ☐ | ☐ | ☐ | ☐ |
| Satisfaction with the specialist | ☐ | ☐ | ☐ | ☐ |
| Overall telemedicine experience | ☐ | ☐ | ☐ | ☐ |

**Total Score:** ______________________

**Would you use telemedicine again?**  ☐ Yes  ☐ No

**Would you recommend telemedicine to someone else?**  ☐ Yes  ☐ No
